# The prevalence and transcriptional activity of the mucosal microbiota of ulcerative colitis patients

**DOI:** 10.1038/s41598-018-35243-4

**Published:** 2018-11-22

**Authors:** Aina E. Fossum Moen, Jonas Christoffer Lindstrøm, Tone Møller Tannæs, Simen Vatn, Petr Ricanek, Morten H. Vatn, Jørgen Jahnsen, The IBD-Character Consortium, Anna B. Frengen, Fredrik A. Dahl, Panpan You, Janne Sølvernes, Gunn S. Ekeland, Trond E. Detlie, Christine Olbjørn, Kate R. O’Leary, Nicholas T. Ventham, Nicholas A. Kennedy, Rahul Kalla, Alex Adams, Hazel E. Drummond, Ray Boyapati, Elaine R. Nimmo, David C. Wilson, Jack Satsangi, Simon C. Heath, Marta Gut, Angelika Merkel, Monica Bayes, Ivo G. Gut, Åsa V. Keita, Johan D. Söderholm, Henrik Hjortswang, Adam Carstens, Daniel Bergemalm, Jonas Halfvarson, Erik Andersson, Mårten Lindqvist, Dirk Repsilber, Marieke Pierik, Daisy Jonkers, Fernando Gomollón, Mauro D’Amato, Leif Törkvist, Fredrik Hjelm, Mats Gullberg, Niklas Nordberg, Anette Ocklind, Erik Pettersson, Daniel Ekman, Mikael Sundell, Eddie Modig, Ferdinando Bonfiglio, Anne-Clémence Veillard, Renaud Schoemans, Dominique Poncelet, Céline Sabatel, Torbjørn Lindahl, Ewa Ciemniejewska, Christina Casén, Charles Lees, Colin L. Noble, Ian Arnott, Gwo-Tzer Ho, Alan G. Shand

**Affiliations:** 1 Department of Clinical Molecular Biology (EpiGen), Division of Medicine, Akershus University Hospital, Lørenskog, and University of Oslo, Oslo, Norway; 2 Health Services Research Unit, Akershus University Hospital, Lørenskog, Norway; 3 Institute of Clinical Medicine, Campus Ahus, University of Oslo, Oslo, Norway; 4 Department of Gastroenterology, Division of Medicine, Akershus University Hospital, Lørenskog, and University of Oslo, Oslo, Norway; 5 Department of Pediatric and Adolescent Medicine, Akershus University Hospital, Lørenskog, Norway; 6 Gastrointestinal Unit, Centre for Genomics and Molecular Medicine, University of Edinburgh, Edinburgh, UK; 7 Department of Child Life and Health, University of Edinburgh, Edinburgh, UK; 8 CNAG-CRG, Centre for Genomic Regulation (CRG), Barcelona Institute of Science and Technology (BIST), Barcelona, Spain; 9 Universita Pompeu Fabra (UPF), Barcelona, Spain; 10 Department of Clinical and Experimental Medicine, Faculty of Health Sciences, Linköping University, Linköping, Sweden; 11 Department of Gastroenterology, Faculty of Medicine and Health, Örebro University, Örebro, Sweden; 12 School of Medical Sciences, Faculty of Medicine and Health, Örebro University, Örebro, Sweden; 13 Division of Gastroenterology-Hepatology, NUTRIM School of Nutrition and Translational Research in Metabolism, Maastricht University Medical Center, Maastricht, The Netherlands; 14 Digestive Diseases Unit, Facultad de Medicina, Universidad de Zaragoza, Zaragoza, Spain; 15 Unit of Gastrointestinal Genetics, BioDonostia Health Research Institute, San Sebastian and Ikerbasque, Basque Science Foundation, Bilbao, Spain; 16 Karolinska Institutet, Stockholm, Sweden; 17 IBD unit, Centre for Digestive Diseases, Karolinska University Hospital, Stockholm, Sweden; 18 Olink Proteomics, Uppsala, Sweden; 19 Diagenode SA, Seraing, Belgium; 20 Genetic Analysis AS, Oslo, Norway; 21 Translational Gastroenterology Unit, Nuffield Department of Medicine, Oxford, OX3 9DU UK; 22 IBD Pharmacogenetics, University of Exeter, Exeter, UK; 23 Gastrointestinal Unit, Western General Hospital, Edinburgh, UK; 24 Q-linea AB, Uppsala, Sweden

## Abstract

Active microbes likely have larger impact on gut health status compared to inactive or dormant microbes. We investigate the composition of active and total mucosal microbiota of treatment-naïve ulcerative colitis (UC) patients to determine the microbial picture at the start-up phase of disease, using both a 16S rRNA transcript and gene amplicon sequencing. DNA and RNA were isolated from the same mucosal colonic biopsies. Our aim was to identify active microbial members of the microbiota in early stages of disease and reveal which members are present, but do not act as major players. We demonstrated differences in active and total microbiota of UC patients when comparing inflamed to non-inflamed tissue. Several taxa, among them the *Proteobacteria* phyla and families therein, revealed lower transcriptional activity despite a high presence. The *Bifidobacteriaceae* family of the *Actinobacteria* phylum showed lower abundance in the active microbiota, although no difference in presence was detected. The most abundant microbiota members of the inflamed tissue in UC patients were not the most active. Knowledge of active members of microbiota in UC patients could enhance our understanding of disease etiology. The active microbial community composition did not deviate from the total when comparing UC patients to non-IBD controls.

## Introduction

Ulcerative colitis (UC) and Crohn’s disease represent two of the most common types of inflammatory bowel disease (IBD). These are complex immune-mediated disorders characterized by chronic intestinal inflammation, where genetics, the environment and the gut microbiota are all factors related to disease initiation and progression^1–3^. The role of the gut microbiota in the pathogenesis of IBD is still poorly understood, despite extensive research efforts during the last several decades. Several studies have revealed a pattern of reduced alpha diversity and abnormal microbial community composition with a decrease in a number of taxa within the phylum *Firmicutes* and an increase in the phyla *Bacteriodetes* and *Proteobacteria*^4,5^. At the species level, some candidates have been reported to have an altered abundance in diseased versus healthy subjects, but a microbial signature of disease has not been found^3,6,7^. The normal, healthy human gut microbiota, seen at the phylogenetic level, exhibits a generally high variability between and within individuals over time^4,8,9^. However, functional redundancy can reduce the effect of variations in microbial community structure, and the healthy gut microbiota is relatively stable at a functional level^8,10^.

16S rRNA gene sequencing is widely used for the determination of the total microbial community composition. A potential drawback in using 16S rRNA gene sequencing is that its results comprise a total mixture of active, dormant and dead bacteria. There are methods available to exclude detection of dead microbes^11–13^. Recent studies have utilized propidium monoazid (PMA) treatment prior to metagenomics sequencing (PMAseq) to exclude dead bacterial cell DNA from further analysis^14,15^. Our study focus on the mucosal microbiota and the PMAseq method cannot be used on stabilized tissue biopsies due to the stabilization process itself and the biopsy sample consistency^16^.

We, together with other researchers, have used the transcribed 16S rRNA counts, as opposed to the gene counts, to indicate the potential bacterial metabolic activity of a sample as rRNA levels correlate with the protein synthesis potential of the microorganisms and hence can be used to estimate their activity^17–19^. The potential active microbial community composition differs from the total microbial community composition in healthy and diseased individuals. The observation that active microbes seem to have a larger impact on gut health status than inactive or dormant microbes highlights the importance of studying metabolically active microbiota in both diseased and healthy individuals^20–22^.

In the present study, we investigated the composition of both the active and total microbiota of newly diagnosed, treatment-naïve UC patients to uncover the microbial picture at the time of disease diagnosis.

The functional capacity of the microbiota was further elucidated using Piphillin^23^. Our aim was to understand which microbial community members are active at diagnosis and which members are present, but may not be major players, in the early stages of the disease.

## Results

Forty-six UC patients and 39 symptomatic non-IBD controls were included in the study, and 114 and 39 biopsy samples were collected, respectively. All the biopsies yielded sufficient DNA and RNA quantities and qualities for further analyses. The DNA and RNA samples were subjected to microbial profiling using 16S rRNA gene and gene transcript sequencing on the Illumina MiSeq platform with 250 bp overlapping paired-end reads. The samples with fewer than 3 000 and 10 000 raw reads, set from the DNA and RNA blanks, respectively, were excluded from further analysis. This cut-off left 137 of the 153 DNA samples and 129 of the 153 RNA samples from 44 of 46 UC patients and 35 of 39 symptomatic non-IBD controls to be further analysed (Tables 1, S1 and S2).

**Table 1:** Characteristics of ulcerative colitis patients and symptomatic non-IBD controls.

	Ulcerative colitis	Non-IBD
N	44	35
Females	22	18
Age, median (range); years	35 (18, 66)	35 (21, 69)
Antibiotics 3 to 13 months before inclusion	1	2
**Disease extent**		
Proctitis (E1)	10	—
Left-sided colitis (E2)	14	—
Extensive colitis (E3)	20	—
**Samples (inflamed/control)**		
Ileum	0/35	0/28
Ascending colon	16/14	0/7
Descending colon	14/18	0/0
Rectum	13/0	0/0

After quality filtering and assembling of overlapping paired-end reads in mothur, more than 7.9 million sequences were retained in both the DNA and the RNA datasets (with a mean of 67 071 per sample).

The sequences were clustered into 2043 OTUs at 97% identity. The OTUs spanned 122 microbial families and 20 phyla. Aggregating the OTU counts into families and phyla for abundance analyses resulted in 26 and 24 families remaining in the DNA and RNA datasets, respectively.

### Small systematic differences in diversity

**Figure 1:**
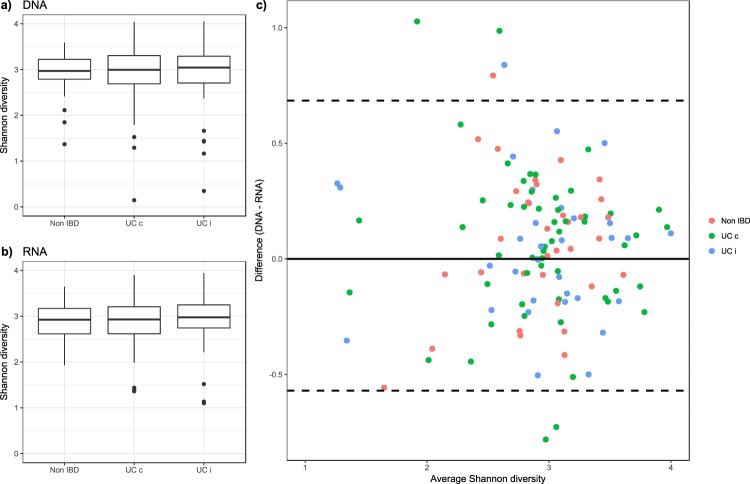
Alpha diversity in inflamed and non-inflamed tissue. Distribution of alpha diversity in the inflamed and non-inflamed samples from UC patients and control patients, showing (**a**) the DNA and (**b**) the RNA datasets, and (**c**) a scatterplot of the alpha diversity in the paired RNA and DNA samples, with the average alpha diversity on the x-axis and the difference on the y-axis.

There was no significant difference in the alpha diversity between the UC patients (inflamed biopsies and non-inflamed biopsies) and the symptomatic controls, when comparing either the DNA or RNA samples (Fig. 1a and b). A non-significant lower diversity of the symptomatic non-IBD control group was observed in the RNA dataset (Fig. 1a). The Bland-Altman plot of the paired RNA and DNA samples showed some heterogeneity in the alpha diversity across the samples but few systematic differences between groups (Fig. 1c). Most of the samples showed a difference within + − 0.5. These differences cannot be explained by the disease status or the inflammation status.

We performed a PCoA analysis with UniFrac distances to investigate whether the sample location could differentiate the samples and thus interfere with further analyses. The results show that alpha diversity is a main factor differentiating the samples and not the sampling location, inflammation status or disease status (Figures S1, S2 and S3).

**Figure 2:**
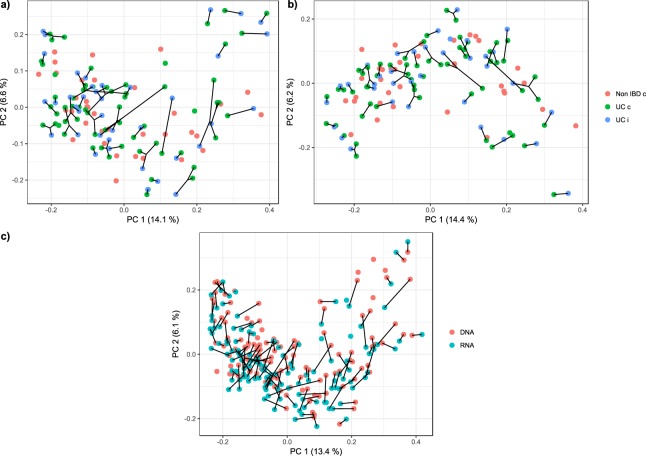
UniFrac distances between different diagnosis groups and datasets. PCoA plot for **(a**) the DNA dataset and (**b)** the RNA dataset. Each sample is coloured according to the disease status and inflammation status. The black lines connect the samples from the same patient. **c)** PCoA plot of a combined analysis from both the DNA and RNA dataset. Paired RNA-DNA samples are connected with black lines.

The PCoA plot of UniFrac distances in the DNA and RNA datasets shows that the biopsy samples from the same patient are similar to each other and that there is no separation between the samples from the UC patients and the symptomatic non-IBD controls (Fig. 2a and b). Investigating the RNA and DNA data from the same samples, these are found near each other in the PCoA plot, indicating an overall pattern of agreement between the two datasets but not complete correspondence (Fig. 2c).

### Taxonomic differences in total and active microbiota between inflamed and non-inflamed mucosa of UC patients

When comparing differences in the total (rRNA gene) microbial community composition between the inflamed and non-inflamed mucosa of UC patients, a significantly higher abundance of *Enterobacteriaceae* (phylum *Proteobacteria*) and a higher but not significant abundance in the *Firmicutes* family *XI Incertae Sedis* and in the *Actinobacteria* family *Actinomycetaceae* were observed in the inflamed mucosa (Tables 2, S3 and S4). However, none of these differences were observed when active (rRNA) microbial community compositions were compared. For the active microbiota, a significantly lower transcriptional activity in the *Actinobacteria* family *Bifidobacteriaceae* and a slightly lower transcriptional activity in the *Proteobacteria* family *Alcaligenaceae* was found in the inflamed mucosa compared to the non-inflamed mucosa. In addition, although not significant, the *Prevotellaceae* showed a higher transcriptional activity (Table S4).

**Table 2:** Top hits of differences between the inflamed and non-inflamed samples in UC patients.

Nucleic Acid	Phyla	Family	Fold Change^†^	p-value
DNA	*Proteobacteria*		1.331	0.010
DNA	*Proteobacteria*	*Enterobacteriaceae*	2.455	0.002
RNA	*Actinobacteria*		0.767	0.008
RNA	*Proteobacteria*		0.846	0.010
RNA	*Actinobacteria*	*Bifidobacteriaceae*	0.563	<0.000
RNA	*Proteobacteria*	*Alcaligenaceae*	0.808	0.004

^†^A fold change >1 indicates a higher abundance in the inflamed samples than in the non-inflamed control samples.

Interestingly, when performing the analysis at the phylum level, a significantly higher abundance of *Proteobacteria* was found in the total microbiota in the inflamed tissues, while a significantly lower transcriptional activity was found in the active microbiota. Also a significantly lower transcriptional activity of *Actinobacteria* was found, and a similar but not significant difference in the abundance in the DNA dataset.

### Taxonomic differences in total and active microbiota of non-inflamed mucosa between UC and symptomatic non-IBD controls

At the phylum level, a significantly lower abundance of *Bacteroidetes* was found in the total microbiota of the UC patients compared to the symptomatic non-IBD controls (Tables 3 and S5). A significantly higher abundance and transcriptional activity was found in *Proteobacteria* in UC patients compared to the symptomatic non-IBD controls in both the total and active microbiota, respectively.

**Table 3:** Top hits of differences between the non-inflamed tissues from UC patients and non-IBD controls.

Nucleic Acid	Phyla	Family	Fold Change^†^	p-value
DNA	*Proteobacteria*		2.675	<0.001
DNA	*Bacteroidetes*		0.844	<0.001
DNA	*Proteobacteria*	*Enterobacteriaceae*	4.473	<0.001
DNA	*Firmicutes*	*Peptostreptococcaceae*	4.205	0.001
DNA	*Bacteroidetes*	*Prevotellaceae*	0.197	0.009
RNA	*Proteobacteria*		2.206	0.004
RNA	*Firmicutes*	*Peptostreptococcaceae*	5.669	<0.001
RNA	*Proteobacteria*	*Enterobacteriaceae*	3.861	0.001
RNA	*Bacteroidetes*	*Prevotellaceae*	0.408	<0.001

^†^A fold change > 1 indicates a higher abundance in samples from UC patients than in non-IBD patients.

Several bacterial families were found to have an increased or decreased fold change (FC) in both the total and active microbiota, but this was not significant at the 0.05 level (Table S6).

### Paired comparisons of total and active microbiota

**Figure 3:**
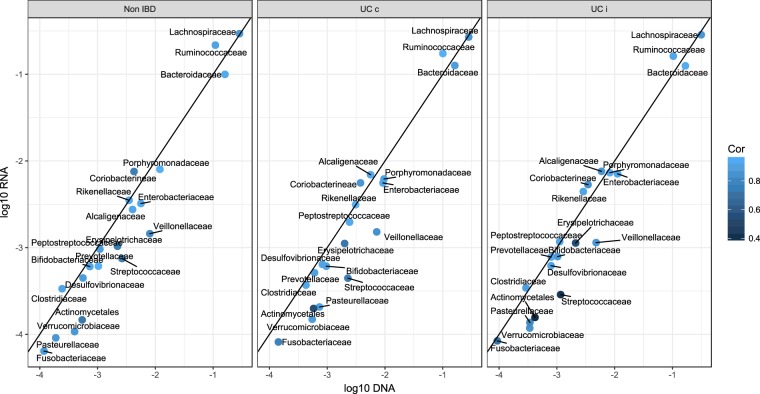
Average log transformed abundances of bacterial families in the total and active microbiota. The diagonal line indicates identical abundances in the DNA and RNA datasets. The colour intensity shows how much the abundance of the bacterial families correlate.

Our nucleic acid extraction method allowed for the direct comparison of total and active microbial community compositions in each biopsy. The transcriptional activity and the total abundances at the family level generally agreed and were highly positively correlated (Fig. 3). A paired Wilcoxon test of family-level abundances showed significant differences for most families, but the only family where the differences were of any meaningful magnitude was *Ruminococcaceae*, with a mean abundance of approximately 10 percentage points higher in the active microbiota (Table S7). These associations hold across disease status and inflammation status.

**Figure 4:**
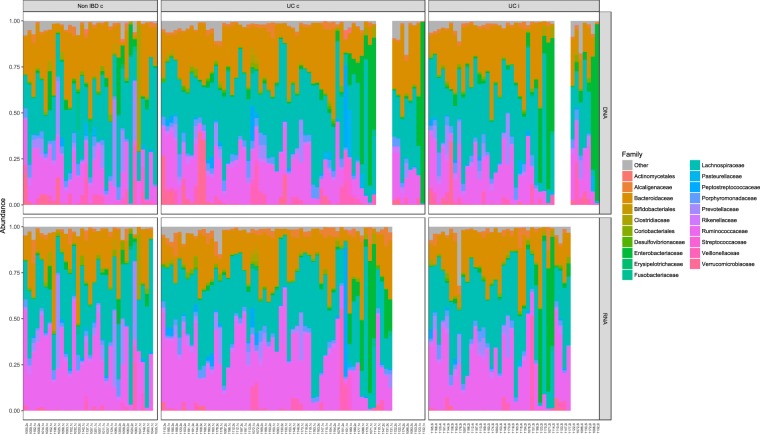
Bacterial taxonomic family abundances in total and active microbiota in all the samples. The three families *Ruminococcaceae*, *Lachnospiraceae* and *Bacteroidaceae* dominated in both datasets.

Comparing the microbial composition on the taxonomic family level in the active and total microbiota of each biopsy, collected from inflamed and non-inflamed tissues from UC patients and symptomatic non-IBD controls, revealed compositional similarities (Fig. 4). The same three families *Ruminococcaceae*, *Lachnospiraceae* and *Bacteroidaceae* dominated in both datasets. Some overall differences were found, such as a larger proportion of active *Ruminococcaceae* compared to total *Ruminococcaceae* in the symptomatic non-IBD controls, and a larger proportion of *Verrucomicrobiaceae* was seen in the total microbiota compared to the active microbiota.

**Figure 5:**
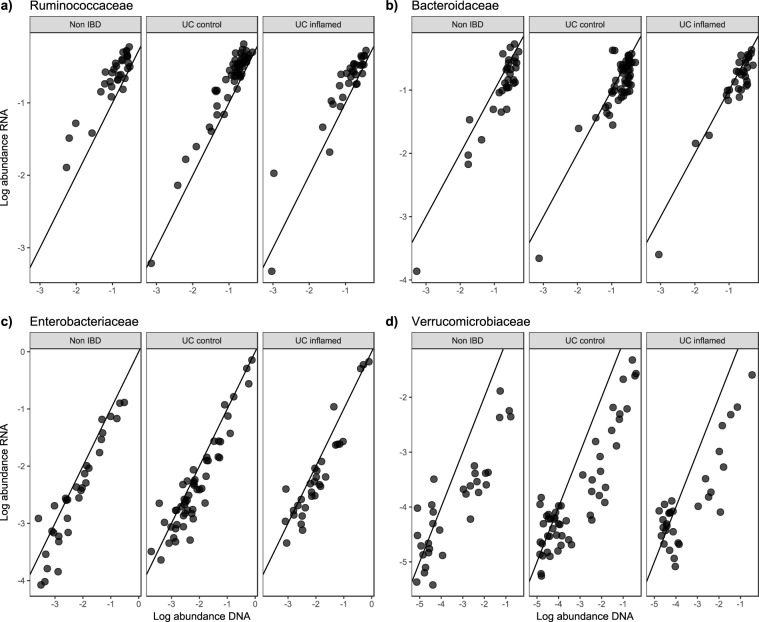
Distribution of total and active microbiota abundances across disease and inflammation status. A subset of the most prevalent and active bacterial families.

In most families, there was a proportional relationship between the total and active microbiota (Table S7). However, some important exceptions were detected (Fig. 5a–d). *Ruminococcaceae* showed increased activity (Fig. 5a), whereas *Bacteroidaceae* and *Verrucomicrobiaceae* were more genetically abundant (Fig. 5b and d). The *Enterobacteriaceae* members, usually reported as important contributors to IBD-associated dysbiosis, were found to be less transcribed, indicating a partially dead or dormant population (Fig. 5c).

### The functional dysbiosis in the mucosal tissue

The Piphillin tool returned a total of 295 KEGG pathway abundances (Tables S8–S11). The functional predictions based on DNA sequencing revealed the following results. Comparing the non-inflamed mucosal samples from UC patients with symptomatic non-IBD patients revealed significant differences among the metabolic pathways of chemical carcinogenesis (ko05201), drug metabolism – cytochrome P450 (ko00982), metabolism of xenobiotics by cytochrome P450 (ko00980) and carotenoid biosynthesis (ko00906), all of which were more present in total microbiota in the UC patients.

The functional analysis based on RNA (cDNA) sequencing did not show any significant differences (Tables S9 and S11).

## Discussion

Our results support the use of RNA as a study tool in microbiota studies. The present study shows that the most abundant bacterial taxa, found in the inflamed and non-inflamed human mucosal tissue microbiota in UC patients, differed from the most active taxa. Using 16S rRNA [11] to reveal the potential active microbial taxa, we found a significant decrease in members of the phylum *Proteobacteria*, while the 16S rRNA gene revealed a significant increase in the total microbiota. Comparing UC patients to symptomatic non-IBD controls revealed more corresponding results between the 16S rRNA and 16S rRNA gene amplicon counts at the phylum level.

In a recent study by Heinsen and colleagues^17^, it was discovered that OTU abundance and potential activity in a bacterial community do not necessarily conform to each other. As such, we aimed to study the active and total microbial members of the colonic mucosal microbiota in treatment-naïve UC patients in search of bacterial taxa that may have a larger pathological role defined by their potential activity. In UC, the colonic mucosal tissue is inflamed, resulting in microbes with direct access to the epithelial layer. We believe that microbes thriving and having a potentially high metabolic activity in this environment will have a more important role in the disease than the total bacterial community occupying the same niche. The mucosal biopsy samples in the present study were subjected to an extensive nucleic acid extraction protocol optimized to extract and preserve DNA and RNA from both hard-to-lyse and more-prone-to-lyse bacterial taxa. This is an important step to obtain a balanced nucleotide purification and thus get a better representation of the microbes present in the samples. The DNA and RNA of each sample were isolated from the same single mucosal colonic biopsy, making it possible to perform a direct comparison between the active and total microbiota^24,25^. Other groups have purified DNA and RNA from single biopsies in their work on microbiota, but the purification methods have not been optimized for microbial nucleic acids^17,26^

Comparing the paired RNA and DNA samples revealed a modest heterogeneity in the alpha diversity (Fig. 1c). The alpha diversity analyses showed no significant differences when comparing the UC patients, the inflamed and non-inflamed tissue, and the symptomatic non-IBD control group in either dataset (Fig. 1a and b). An explanation for the lack of differences in alpha diversity and the lack of large differences in the active and total microbial community compositions when analysing both within UC patients and between UC patients and symptomatic non-IBD controls could be the result of mixing disease severity in our study group (Table S1). Walujkar^27^ and colleagues found in their study different shifts in the mucosal microbiota following UC severity stages. In the present study, we included patients suffering from different degrees of severity. Possible shifts in the diseased microbiota could be masked due to this mixing, hence shielding differences within the patient group. The control group of symptomatic non-IBD patients included patients suffering from IBD-like symptoms but not experiencing tissue inflammation. This patient group will most likely have a disturbed microbiota but probably to a lesser extent than the IBD patients. Thus, the analyses between the UC patients and the symptomatic non-IBD control group could also be affected by the mixing of disease severity in the former group.

Overall composition analyses with UniFrac showed that between-patient differences were more important than other potential sources of differentiation, such as diagnosis status and inflammation status (Fig. 2a and b). Pairwise differences between the active and total microbiota were also small (Fig. 2c).

To compare the mucosal microbiota between UC patients and symptomatic non-IBD controls, non-inflamed tissue biopsies were collected in the terminal ileum or the ascending colon if the terminal ileum could not be reached. This sampling area was chosen in an attempt to use data from all the UC patients, including the total colitis patients. As the sampling area of the non-inflamed tissue biopsies varied between the two parts of the intestine, analyses were performed to investigate whether the sampling could affect the further microbiota analysis. The analyses did not reveal any significant difference in either the 16S rRNA gene or the transcribed 16S rRNA counts between the non-inflamed tissues sampled from different gut locations within the patients (Fig. S1). Furthermore, our analysis revealed that even inflamed and non-inflamed tissue samples within the same patient were more similar with regard to both total and active microbiota than microbiota from the same location and with the same inflammation status in other patients (Fig. 2). These findings support other studies, indicating that there is greater variability between subjects, regardless of diagnosis, location in the gut and inflammation status, than the internal variability^1^.

Analyses of the inflamed and non-inflamed mucosal microbiota in UC patients revealed a greater abundance of *Proteobacteria* in the total microbial community composition of the inflamed tissues, with an FC of 1.331 (Table 2). This finding was contrasted by the active microbial community composition, which showed a lower transcriptional activity in the inflamed tissues, with an FC of 0.846. The *Proteobacteria* family level revealed a 2.455-fold higher abundance in *Enterobacteriaceae* of the total microbiota. The active microbiota was not found to have a significantly higher transcriptional activity, indicating that *Enterobacteriaceae* might not have an increased function or a more pathophysiological role in the inflamed tissue compared to the non-inflamed tissues of UC patients. The pairwise comparison within biopsies supported this finding, with less transcriptional activity of the bacterial family in the RNA dataset and higher abundance in the DNA dataset (Fig. 5c). In the active microbiota, a 0.808-fold lower transcriptional activity was found in the *Proteobacteria* family *Alcaligenaceae*. This result was not found in the total microbiota. The contrasting difference in abundance and activity in tissues with different inflammatory statuses may indicate that *Proteobacteria* have important pathophysiological functions in UC patients, being more abundant and active compared to symptomatic non-IBD controls, but do not have any increased function in the area of the diseased tissue. The discrepancies observed between the active and total microbiota could possibly be explained by the increased activity of human immune system in inflamed tissue.

The active microbiota showed a lower transcriptional activity in the phylum *Actinobacteria*, with a 0.563-fold decrease in the family *Bifidobacteriaceae*, suggesting a less prominent functional role of this bacterial family in the inflamed mucosa of UC patients. The total microbiota did not show this finding. The lower transcriptional activity could be related to the observed higher transcriptional activity of the aerotolerant anaerobic, saccharolytic, H_2_-producing, mucus-associated *Prevotellaceae* family, for which *Prevotellaceae* may possibly occupy the niche of *Bifidobacteriaceae*.

The paired biopsy analyses showed that at the family level, the active and total abundances generally agreed, but *Ruminococcaceae* had a larger transcriptional activity than its abundance for both the UC patients, regardless of inflammation status of the tissue samples, and the symptomatic non-IBD controls (Figs 4 and 5a).

Our results contrast with the study of Forbes and colleagues^28^, which found no significant variations in any phyla when comparing inflamed and non-inflamed mucosa within a group of UC patients. Reasons for this discrepancy could be differences in the protocol of DNA purification, including different enzymes used in the lysis step, as well as the use of different regions of the variable 16S rRNA gene for next generation sequencing (V5 versus V4, in our study).

The controls in our study were symptomatic non-IBD controls who had predefined symptoms of IBD but did not meet the diagnostic criteria. Elucidation of this group was not performed in the IBD-Character study, but the group probably consists of several patients who were classified as IBS-like. *Proteobacteria* has been found to have a higher abundance in UC patients compared to healthy individuals^29^, but the relation to IBS patients has not been convincing^30^. In the present study, the *Proteobacteria* phylum in the UC patient group, for both the total and active microbiota, revealed a considerably higher abundance and transcriptional activity compared to the symptomatic non-IBD control group (Table 3). Both the total and active microbiota revealed corresponding patterns with regard to significant differences in the fourth- to fifth-fold greater presence of the *Proteobacteria* family *Enterobacteriaceae* and the *Firmicutes* family *Peptostreptococcaceae*, indicating an important contribution of these two families to the diseased microbiota. Both bacterial families have been associated with disease and UC^1,31–33^, and they seem to have a greater function in the mucosal tissue of UC patients compared to the diseased non-IBD patients. Both bacterial families have been found to participate in xenobiotic/drug metabolism^34^, which is also a functional pathway found to be more present in UC patients in the present study. *Enterobacteriaceae* is a family of facultative anaerobes with an increased ability to adhere to the intestinal mucosa and have diverse energy sources. *Peptostreptococcaceae*, on the other hand, is a family of strict anaerobes with a fermentative metabolism. The latter family contains saccharolytic members and has been linked to colon cancer, but its function in the gut microbiota is still largely unclear^33^. The results of the functional analyses revealed a significantly greater abundance in the chemical carcinogenesis pathway in UC patients. UC is a well-known risk factor for colon cancer, and the results hint at a possible role for bacteria in modulating cancer risk^35^.

The *Bacteroidetes* family *Prevotellaceae* was found to be significantly less present and to have a lower transcriptional activity in the UC patients^36^. This is a dysbiosis pattern, seen when comparing UC patients to healthy controls, supporting the view of a less dysbiotic microflora in possible IBS patients^30^.

The pairwise analyses from non-IBD patients revealed, like the analyses of the UC patients, a high presence of the *Clostridiales* family *Ruminococcaceae* in the active microbiota and a low presence in the total microbiota. Schirmer and colleagues^22^ found that *R*. *gnavus* showed a large increase in transcriptional activity in Crohn’s disease and UC patients compared to non-IBD controls. In the present study, changes at the species level could not be detected, thus leaving this possible variation undetected.

This study has limitations. Strict ethical regulations in Norway hinder the collection of ileocolonic biopsies from healthy individuals. For this reason, our control group was comprised of symptomatic non-IBD controls who may have also suffered from a disturbed microbiota, although likely to a lesser extent than UC patients.

In conclusion, we find support for the use of RNA as a study tool in microbiota studies. Knowledge of the active members of microbiota in UC patients could aid in the understanding of the disease ethology. With the mucosal microbiota being in direct contact with the host, the active members could be of particular importance. In the present study, the most abundant microbiota members of the inflamed tissue in UC patients were not the most active. The active microbial community composition did not deviate from the total microbial community composition when comparing UC patients to non-IBD controls; thus, the choice of which microbiota to study depends on the questions raised.

## Material and Methods

### Study population and sample collection

Treatment-naïve UC patients were included at the time of disease diagnosis. The clinical data were collected at the time of inclusion (Table S1). Correspondingly, the patients referred for colonoscopy because of suspected IBD, but who had a normal endoscopic investigation and no elevated faecal calprotectin (Bühlmann Laboratories AG, Basel, Switzerland), were included as symptomatic controls. Celiac disease was excluded by negative serology. Suspected IBD was defined by the presence of predefined symptoms, including abdominal pain, diarrhoea and/or blood in the stool for more than 10 days^37^, with no evidence of enteric infection (Table S1). In the present study, we selected all the available symptomatic controls and an excess of UC patients, who were gender and age matched to the controls. All the patients were recruited from the Akershus University Hospital in Lørenskog, Norway and the study population was part of the EU study IBD-Character (EU ref no 305676) (http://www.ibdcharacter.eu/).

Prior to colonoscopy, the subjects went through bowel cleansing with Picoprep (Ferring Legemidler AS, Oslo, Norway), according to the manufacturer’s instructions. Colonic mucosal biopsies of 2–3 mm in size were collected from patients during colonoscopy at the time of inclusion. For the UC patients, biopsies from macroscopically inflamed tissue and non-inflamed tissue were collected from the same segment, if possible, in addition to a control sample from the ascending colon or the terminal ileum, if accessible. From the non-IBD control group, biopsies were collected from the ascending colon or the terminal ileum, when possible. All the biopsies were placed in Allprotect Tissue Reagent (Qiagen, Hilden, Germany) and stored according to the manufacturer’s instructions.

### Ethics approval and consent to participate

The study, including data collection and analyses, was approved by the Regional Committee for Medical Research Ethics, South-Eastern Norway, reference no. REK sør-øst 2009/2015 and the representative of privacy protection at Akershus University Hospital, reference no. 13–126. All experiments were performed in accordance with and following the Declaration of Helsinki Principles. All methods were performed in accordance with the relevant guideline and regulations. All patients gave their written informed consent prior to colonoscopy and their inclusion in the IBD-Character study.

### Nucleic acid purification

The microbial RNA and DNA were purified using the AllPrep DNA/RNA Mini kit (Qiagen) following a modified protocol published by our group previously^24^. In short, the biopsies were subjected to a thorough lysis and homogenization procedure, involving both enzymatic and mechanical lysis steps. This procedure ensured the protection of RNA by removing the lysate after each homogenization step. The DNA and RNA were eluted using 40 µl RNase-free water and were stored at -20 °C and -80 °C, respectively. The concentrations of the DNA and RNA samples were assessed using a NanoDrop ND-1000 spectrophotometer (Thermo Fisher Scientific, Waltham, MA, USA) and using the OD_260_ for calculation. For the DNA, the quality was obtained using the OD_260_/OD_280_ and OD_260_/OD_230_ ratios for purity assessment of the samples. The RNA quality, indicated by the RNA integrity number (RIN), was assessed with an Agilent 2100 Bioanalyser, Agilent 2100 Expert software and Agilent RNA 6000 Nano Kit (Agilent Technologies Inc., Santa Clara, CA, USA).

### cDNA synthesis

The cDNA was synthesised from 1 µg RNA from each sample using the AccuScript High Fidelity 1st Strand cDNA Synthesis Kit (Agilent Technologies Inc.) and random hexamers, according to the manufacturer’s instructions. Two random RNA samples were run in the absence of reverse transcriptase to assess the degree of contaminating genomic DNA.

### 16S rRNA and 16S rRNA gene amplicon sequencing

Two 16S amplicon libraries, consisting of a 16S rRNA gene and 16S rRNA transcript, were made according to a protocol published by Kozich and colleagues^38,39^. In short, the genomic DNA and cDNA were subjected to 16S PCR amplifications of the v4 gene region using a 500 ng and 250 ng template, respectively. The AccuPrime ^TM^ Pfx Supermix (Agilent Technologies Inc.) concentration, PCR primer combinations and thermal cycling conditions described in the MiSeq Wet Lab SOP^39^ were followed, with the exception of the annealing temperature and extension step, which were set to 50 and 68 °C, respectively. The two amplicon libraries were semi quantified using 1% agarose gel quantification and pooled according to band intensity into three pools of high, medium and low concentrations. From each library pool, 200 µl was purified by a 2% agarose gel using QIAquick Gel Extraction Kit (Qiagen) to reduce non-specific amplification products and ensure the removal of primer dimers. The purified products were quantified using the Kapa Library Quantification Kit (Universal) (Kapa Biosystems Inc., Wilmington, MA, USA) and finally pooled according to the concentrations and number of samples. The sequencing was performed using the Illumina MiSeq platform (Illumina Inc., San Diego, CA, USA) and the MiSeq reagent kit v/2 (500 cycles) according to the manufacturer’s instructions, with the addition of custom sequencing primers and index and 8% PhiX, as described in the MiSeq Wet Lab SOP^39^.

### Data analysis

Blank samples were run through the wet lab procedure, from the nucleic acid purification step through the sequencing process, to detect possible reagent contamination. The minimum numbers of reads from each sample was set from the number of reads on the DNA and RNA blanks. MiSeq Reporter software (Illumina Inc.) was used for demultiplexing the reads and for fastQ file generation.

#### Sequence processing

The total sequence data were processed with mothur v.1.36.1 according to the MiSeq SOP^38^. The processed sequences were clustered into operational taxonomic units (OTUs) at 97% identity, and the SILVA reference database release 119 provided taxonomic information. The most abundant sequence in each OTU was picked as the representative sequence for the purpose of downstream sequence-based analyses, including UniFrac distances and functional analyses.

#### Diversity and abundance analysis

OTU-based analyses were done in R^40^. The phyloseq package was used for further data handling, diversity, and ordination analyses^41^. UniFrac distances and Principal Coordinates Analysis (PcoA) were used in the ordination analyses.

A differential abundance analysis was completed at both the family and phylum taxonomic levels. Lower taxonomic levels were not used due to known limitations in performing species-level identification from 16S rRNA sequencing^42^. For each analysis, the OTUs that shared taxonomic rank at each level were merged into one. The number of reads was modelled using a Negative Binomial regression model^43^. When we compared the inflamed and non-inflamed samples in UC patients, we included a random intercept to account for the paired samples. If several non-inflamed samples from the same patient were available, we compared the biopsies taken closest to the inflamed biopsy only. In the comparison between the symptomatic controls and the UC patients, we used the non-inflamed biopsy taken closest to the ileum.

OTUs with an average abundance of less than 0.01% reads and patients who had used antibiotics in the last 13 months were excluded from these analyses. The regression models also included a term for the sample gut location and a scaling factor to account for the total number of reads in the sample. The lme4 R package was used to fit the random effects models, while the MASS R package was used to fit the model without the random effects.

Differences in the diversity indices were investigated with linear regression models, with the same predictor variables as in the abundance analysis.

Paired analyses of the total and active microbial community composition from the same sample was completed using paired Wilcoxon tests on all the families.

#### Functional analyses

To investigate the functional capabilities of the total and active microbiota, we used Piphillin to infer KEGG (version 78.1, May 2016) pathway abundances^23^. Differential abundance analyses were performed in the same manner as in the OTU-based analyses.

## Electronic supplementary material

Supplementary Materials

Table S1

Table S2

Table S3

Table S4

Table S5

Table S6

Table S7

Table S8

Table S9

Tabls S10

Table S11

## Data Availability

The fastq files generated during the current study are available from the corresponding author on reasonable request.
