# Supplementary material for: The prevalence and transcriptional activity of the mucosal microbiota of ulcerative colitis patients
Source: Sci Rep. 2018 Nov 22;8:17278. doi: 10.1038/s41598-018-35243-4 (PMC6250705; doi:10.1038/s41598-018-35243-4)
Supplement: Supplementary file 1 — Supplementary Materials [file 41598_2018_35243_MOESM1_ESM.pdf]

Supplementary Materials

The prevalence and transcriptional activity of the mucosal microbiota of ulcerative colitis patients

Aina E Fossum Moen, Jonas Christoffer Lindstrøm, Tone Møller Tannæs, Simen Vatn, Petr Ricanek, Morten Vatn, Jørgen Jahnsen and the IBD-Character Consortium.

Figures S1 to S3

Supplementary data – Tables S1, S2, S3, S4, S5, S6, S7, S8, S9, S10 and S11

Legends for the supplementary excel format tables:

Supplementary Table S1. Metadata of the 79 included patients with at least one sample passing sequencing quality control.

Supplementary Table S2. Overview of the 145 biopsies that yielded 137 DNA samples and 129 RNA samples passing sequencing quality control including DNA and RNA concentrations, quality and purity measures.

Supplementary table S3. Taxonomic differences in total and active microbiota at phylum level between inflamed and non-inflamed mucosa of UC patients.

Supplementary table S4. Taxonomic differences in total and active microbiota at family level between inflamed and non-inflamed mucosa of UC patients.

Supplementary table S5. Taxonomic differences in total and active microbiota at phylum level of non-inflamed mucosa between UC patients and symptomatic non-IBD controls.

Supplementary table S6. Taxonomic differences in total and active microbiota at family level of non-inflamed mucosa between UC patients and symptomatic non-IBD controls.

Supplementary table S7. Wilcoxon paired comparisons of total and active microbiota at family level.

Supplementary table S8. Piphillin functional predictions of total microbiota comparing inflamed and non-inflamed mucosa of UC patients.

Supplementary table S9. Piphillin functional predictions of active microbiota comparing inflamed and non-inflamed mucosa of UC patients.

Supplementary table S10. Piphillin functional predictions of total microbiota comparing non-inflamed mucosa between UC patients and symptomatic non-IBD controls.

Supplementary table S11. Piphillin functional predictions of active microbiota comparing non-inflamed mucosa between UC patients and symptomatic non-IBD controls.

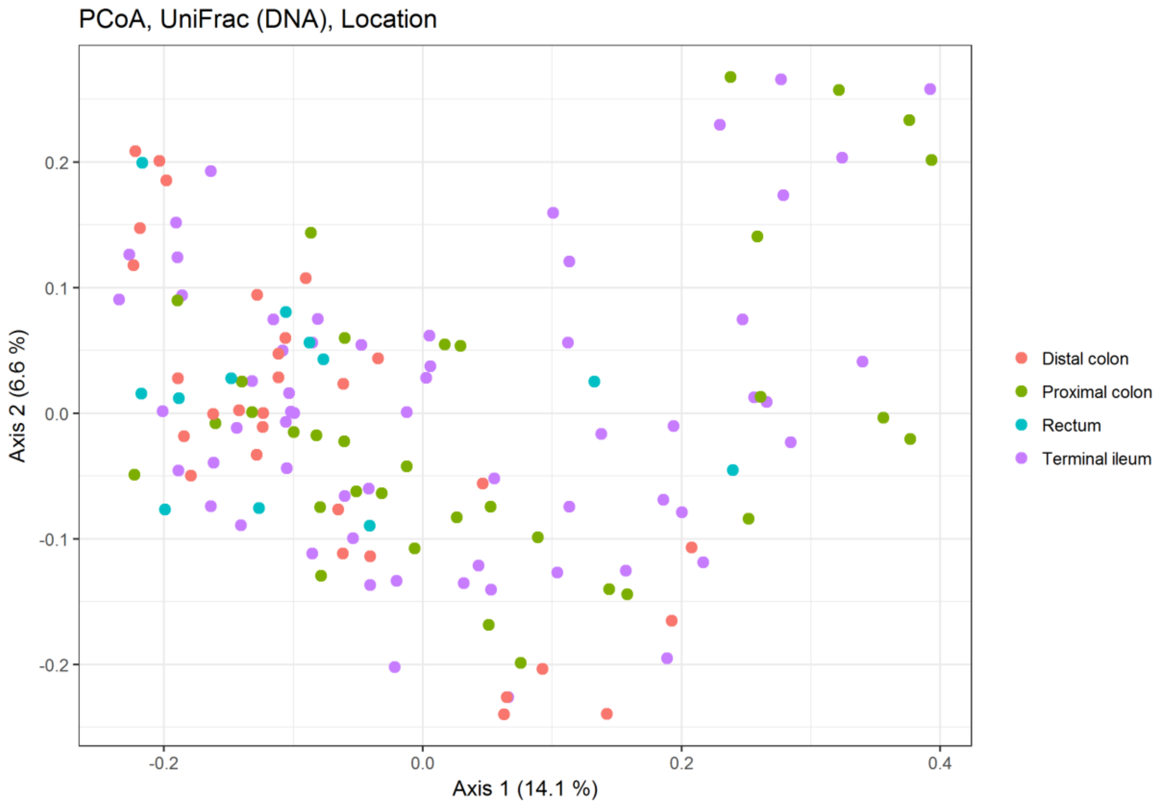

Supplementary Figure S1. UniFrac distances between different biopsy locations for the DNA dataset.

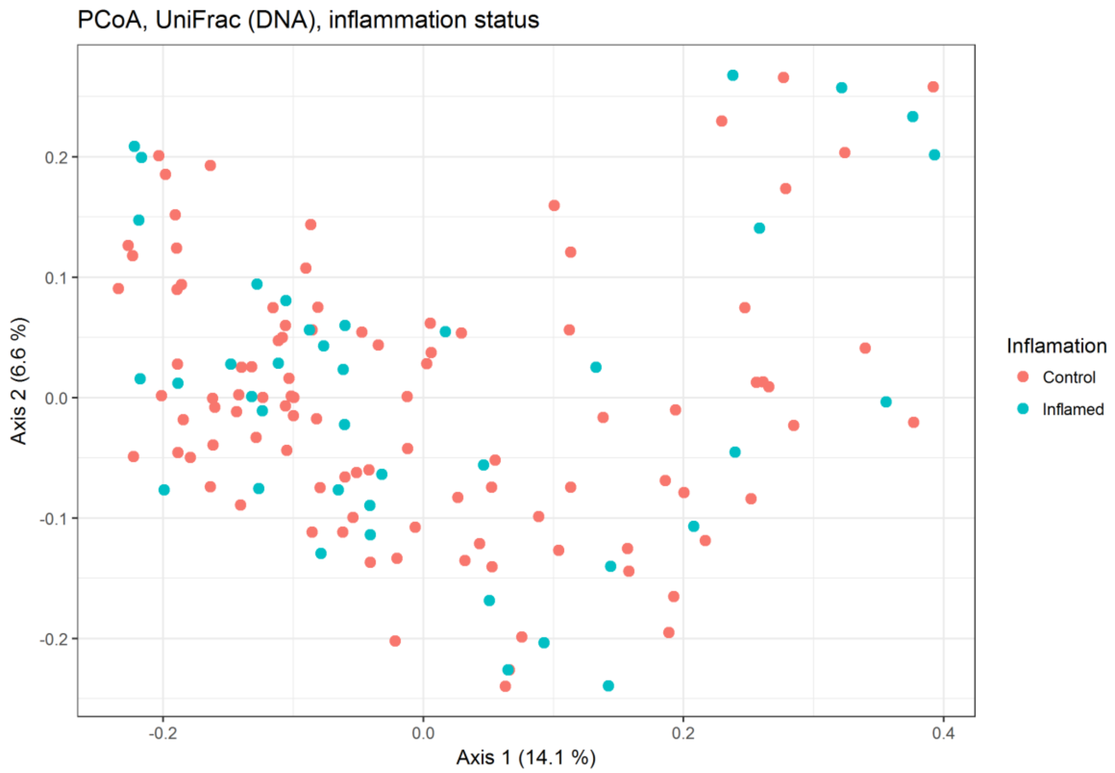

Supplementary Figure S2. UniFrac distances between inflamed and non-inflamed biopsies for the DNA dataset.

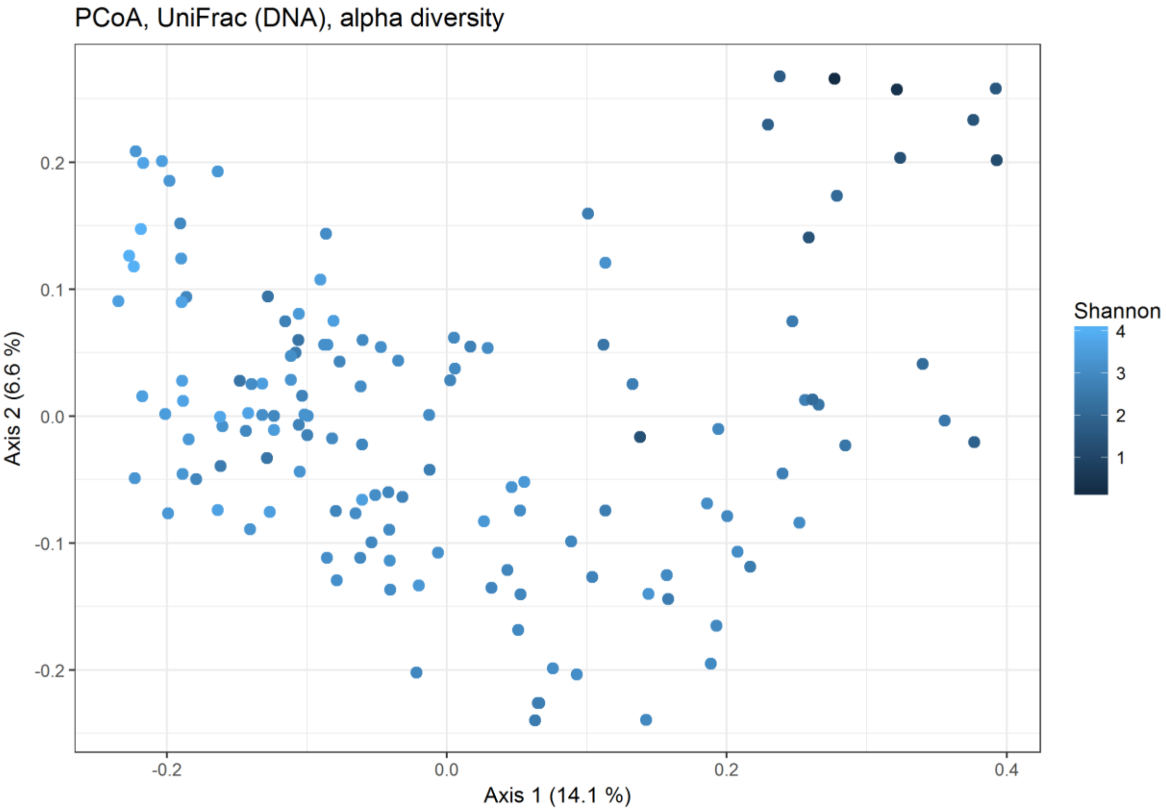

Supplementary Figure S3. UniFrac distances explained by alpha diversity for the DNA dataset.
